# Development of a series of patient information leaflets for constipation using a range of cognitive interview techniques: LIFELAX

**DOI:** 10.1186/1472-6963-7-3

**Published:** 2007-01-04

**Authors:** Amelia A Lake, Chris Speed, Anna Brookes, Ben Heaven, Ashley J Adamson, Paula Moynihan, Sally Corbett, Elaine McColl

**Affiliations:** 1 Human Nutrition Research Centre, School of Clinical and Medical Sciences & Institute of Health and Society, Newcastle University, Framlington Place, NE2 4HH, Newcastle, UK; 2 Institute of Health and Society, Newcastle University, 21 Claremont Place, NE2 4AA, Newcastle, UK; 3 Child Dental Health, School of Dental Sciences, Newcastle University, NE2 4BW, Newcastle, UK; 4 Northumbria Healthcare NHS Trust, North Tyneside General Hospital, Rake Lane, NE29 8NH, North Shields, UK

## Abstract

**Background:**

The aim of the LIFELAX randomised controlled trial (diet and lifestyle vs. laxatives in the management of chronic constipation) is to develop and evaluate a cost effective intervention to promote diet and lifestyle in the treatment and management of chronic constipation for older people in Primary Care. Constipation affects the quality of life in around 20% of older people in the community. In the 65 years plus population, a significant proportion of men and women both living in institutions (81% and 75% respectively) and free living (30% and 37% respectively) use laxatives.

Approximately £42 million is spent each year on prescribed laxatives in England in addition to laxatives purchased over the counter. Although bowel problems are often multifactorial, diet and lifestyle have an extremely important role in their management. This paper describes one aspect of the main study, the development and piloting of the Patient information leaflets (PILs).

**Methods:**

Following review of the literature and interviews with practitioners and patients, 8 PILs were designed on: constipation, activity, bowel health, fruit and vegetables, fibre, fluid, alternative therapies and laxatives. To check the patient's understanding of terms used in the PILS and the clarity and accessibility of the information understanding, cognitive interviews (CI) were used with nine patients (selected from 3 GP surgeries), aged ≥ 55 years, who had received ≥ 3 prescriptions of laxatives over 12 months. Interviews were recorded and transcribed.

**Results:**

Changes made following the CI process included the lay-out, words used (e.g. 'exercise' was changed to 'activity', 'gut motility' changed to 'bowel movement') and descriptions and examples were adapted to be more appropriate for the target population.

**Conclusion:**

Pilot testing with CIs resulted in improvements in the PILs, which emphasises the need to pilot PILs with the target population before use. The techniques employed are relatively inexpensive and could be routinely used when preparing literature for research or clinical use including those intended for use with healthcare professionals and patients.

## Background

This paper describes the first developmental phase of the LIFELAX study (diet and lifestyle vs. laxatives in the management of chronic constipation), which plans to develop and evaluate a cost effective intervention to promote diet and lifestyle in the treatment and management of chronic constipation amongst older people in primary care. Phase 1 involved the development, piloting and refinement of patient information leaflets (PILs) for use in the intervention component of the LIFELAX trial.

Constipation affects quality of life in around 20% of older people in the community [1]. In the U.K. 65 years plus population, a significant proportion of men and women both living in institutions (81% and 75% respectively) and free living (30% and 37% respectively) use laxatives [2]. Prescribed laxatives cost ~£42 million each year in England [3] in addition to those purchased over the counter. Potentially, diet and lifestyle have an extremely important role in the management of chronic constipation.

There is an undisputed need to provide patients with high quality, accurate information [4]. This has potential not only to be beneficial for patients but also for health care providers [4]. Information is a central factor in healthcare [5]. Earlier pilot work from a parallel study, looking at the Stepped Treatment of Older adults On Laxatives (STOOL), found there was a lack of written patient literature relating to constipation for both patients and healthcare professionals in primary care. Those materials which were available were, in the main, produced by pharmaceutical companies and linked to laxative products. The STOOL pilot work also showed differences and inconsistencies between patients and healthcare practitioners with respect to the 'language' and definition of constipation. Use of matched vocabulary, during a consultation, affects patient satisfaction in terms of distress relief, rapport, communication comfort and compliance [6]. The materials produced for LIFELAX aimed to assist participant and health care practitioner communication during the course of the trial.

The approach described here, including the use of plain English in the production of readable and understandable information, and taking into account the views of patients, carers and healthcare professionals, reflects authoritative guidelines [7,8]. To provide specific information about the role of diet and lifestyle in relation to constipation, eight PILs were developed by a dietitian and members of the LIFELAX study team. The LIFELAX PILs were designed to be used within a Behaviour Change Counselling (BCC) model [9]; a client-centred approach which encourages patients to make their own decisions about personal behaviour change. As well as being a source of accessible information, the PILs were designed to enable respondents to think about their current practice and to compare this with recommendations. For example, the PILs on 'Fluid and constipation' contained a fluid counter, the 'Fibre and constipation' PILs contained a fibre counter and the 'Activity and constipation' PILs, an activity counter.

Cognitive interviewing was developed by psychologists and social scientists to reduce sources of response error in survey instruments [10], to identify cognitive response problems with self-reported questionnaires and to improve the quality of data collected [11]. The cognitive interview methods described in this paper have their foundations in techniques pioneered in the mid 1980's in the United States as diagnostic tools for pre-testing questions and questionnaires. The methods lend themselves to pre-testing any written materials including PILs as described here. Two main methods used are "think aloud" interviewing (respondent led – where participants are required to verbalise their thoughts as they are presented with and asked to read new material) and "probing" (interviewer led – direct questions about comprehension, recall, judgement etc are asked). Think aloud is a difficult technique for participants to master. In these circumstances "probing" techniques often lead to an easier interview and provide more data.

Asking individuals to think about their behaviour in relation to the information provided in PILs requires them to engage in a number of cognitive processes. They must first perceive and attend to the information presented in the PIL. They must then read and comprehend that information; the goal is for the reader to understand and interpret the information in the way that the developer of the materials intended – in other words, to share the same meaning. Testing the understanding of PILs involved cognitive process and merited the use of cognitive testing techniques to improve the quality of the information. The method of cognitive interviewing allows for the testing of comprehension and is suitable to test respondent's comprehension of PILs.

In order to design effective PILs, the characteristics of PILs should be evaluated in context to test their suitability for their intended and actual use [12], especially if they are to be used without the presence of a healthcare professional to clarify the contents. It is known that healthcare professionals use of medical labels can have an impact in consultations [13] and can influence patient outcomes [14]. Matching language and ensuring patient understanding is likely to have an impact on patients' understanding of information exchanged and given.

By using a range of cognitive testing techniques, it was possible to 'tap into' the processes and strategies used by respondents whilst thinking about their health behaviour in relation to the information provided in the PILs and to test comprehension of the materials.

The overall objective of this phase of the LIFELAX study was to develop PILs to be used in delivering the diet and lifestyle intervention in the LIFELAX RCT. Here, the process of developing and testing multiple PILs for a specific patient population is explained, with particular focus on the application of cognitive interviewing techniques to test the clarity and suitability of the information.

This paper explores a group of patients' awareness and understanding of the condition of constipation and reports on two core aspects of this developmental process:

• The process of developing and piloting a set of PILs

• The advantages and importance of using cognitive interviewing techniques as a method for developing PILs

## Methods

An earlier exploratory qualitative study (STOOL) conducted interviews with nine GPs and seventeen nurses working in primary care about their experiences of treating and managing older people with constipation. A comprehensive literature review was undertaken to explore the evidence for diet and lifestyle interventions in relation to constipation. Every effort was made to ensure advice given in the PILs was evidence based. In addition, multi-disciplinary experts in the field of constipation, nutrition and primary care were consulted, to ensure quality and suitability of the PILs. Eight PILs were developed on the themes of: Constipation, Bowel health, Laxatives, Fibre, Fluid, Fruit & Vegetables, Activity and Alternative Therapies. Health professionals could then use the PILS specific to the patient's individual requirements. Three of the PILs were interactive and allowed patients to enter personal information and compare this to recommended guidelines. The PILs were designed as A5 booklets, which contained both text and relevant images. Before the PILs were cognitively tested readability scores on the text were conducted using Microsoft Word. Readability tests are based on factors such as word and sentence length, but it is accepted that these scores may be of limited value in the testing of PILs [8]. The readability score does not account for any other features of the PILs such as layout or understanding and was deemed to be insufficient as a testing tool on its own.

Fifty patients from two GP practices who were aged 55 years and over and had received three or more prescriptions of laxatives in the last 12 months (and thus likely to be experiencing chronic constipation) were approached by letter from their GP and invited to take part in the pilot study. Patients were excluded if; they were resident in long-term care. Had inflammatory bowel disease, intestinal obstruction/bowel strictures, known colonic carcinoma, and conditions contra-indicative to the prescription of laxative preparations. Were unable to read and understand written treatment plans and educational written treatment plans and educational material. Ethical approval was obtained from the Local Research Ethics Committee and all participants provided written informed consent.

Cognitive interviews were conducted by the researchers (AL and AB) in the patient's home and recorded on minidisk, with contemporaneous field notes. Depending upon the length of the PILs, either one or two were shown to the patient at each interview. The cognitive interviews employed a probing technique [10], that requires the interviewer to use mainly open questions to explore the participant's understanding of the leaflet. As illustrated by Table 1, the probing technique was interviewer led and the respondent was asked direct questions relating to their comprehension of the material in the PILs. In addition, respondents were asked to paraphrase statements from the PILs or to offer alternative words. Patients were asked about the appearance of the PILs (layout, text size, illustrations) and about specific examples used in the text (Table 2).

**Table 1:** Examples of questions used in the cognitive interviews

From looking at the front cover – the writing and pictures, what do you think this leaflet is going to be about?
What's your understanding of the word: Bowel, Gut, Stimulate, Motility, Intestine, Friendly bacteria, Digestive system, Lactose, Fibre, Abdomen, Senna, Emotional/psychological upset, Prebiotics, Probiotics, Dehydrating.
What do you associate with: Being active Fluids/soft drinks
Would you know where to find this information on a food product?
What do you understand by this sentence?
When you read this sentence what are you thinking?
How would you put this sentence in your own words?
What are you thinking while you read this?
What's this sentence telling you?
Can you explain this sentence to me?
Do you understand how to fill in the fibre/fluid/activity counters? Can you have a go at filling them in?
What will stick in your mind from this leaflet?
How easy (difficult) did you find this question to answer?

**Table 2:** Examples of specific questions used in the interview

What are your overall thoughts about the leaflet?
How could the leaflets be improved?
Is there anything else you would like to see included in the leaflet?
What else do you eat that is not included in this list?
What do you do to relax/be active that is not included in this list?
Would you try any of these ideas?

The interview was both concurrent and retrospective depending on the techniques and probes used. For example, concurrent probing was used as the patient was working on the sheet (e.g. 'what does the picture mean to you as you are reading the front page?'). While retrospective probing was used after the patient had worked on the sheet (e.g. 'when I handed you the leaflet and you first looked at it, what was the most important feature of the front page?').

The recorded interviews were transcribed verbatim. The notes and transcripts were reviewed using content analysis [15] to identify areas of misunderstanding and where modifications to content, wording, layout were indicated. The coding framework was refined and stabilised by inter-researcher agreement and discussion following two independent analyses of the transcripts. QSR N5 NUD*IST (2000 QSR International Pty Ltd. ABN 47 006 357 213 Australia) software was used.

Based on these findings, the PILs were modified; those which caused the most confusion were retested in the same way until the leaflet ceased to cause confusion.

## Results

Nine (7 female, 2 male) respondents consented and completed the study.

### Readability

For the laxative leaflet the Flesch Reading Ease Score (available on Microsoft Word) increased from 64.9 to 70.7, indicating that the leaflet moved towards easier readability.

### Definitions

The respondents' comprehension of specific scientific or biological words was explored by asking for their interpretation of the target word. In describing constipation the words 'gut', 'bowel' and 'intestines' were originally used inter-changeably within the PILs. The cognitive interviews established that the best understood word with this target group was 'bowel', and PILs were changed to reflect this.

When asked to define 'bowel movement' respondents focused on different aspects from straining to texture:

"to have a good performance, like your bowel movement should be not hard"

Issues were raised about the definition of 'normal' bowel movements – which was described in the final version of the PILs as:

'Individuals vary in how often they open their bowels – it ranges from between three times a day and three times a week. So don't be anxious if you don't pass a daily stool. A stool should be solid and easy to pass.' (LIFELAX 'Constipation' PIL)

The concept of 'normal' bowel habits was a difficult issue for the respondents to grasp, as this was pivotal in their individual definition of their constipation. Respondents associated normal bowel habit with being regular and some described their own routine of trying to go to the toilet every morning. In relation to their bowel habits three respondents mentioned that laxatives, stimulants or high fibre foods made their bowel movements more regular. Other respondents defined a normal bowel habit hinting at stool texture and straining. One respondent recognised that it is normal not to have a daily bowel movement, whereas another respondent slightly despairingly said:

"I don't know what normal is".

Another respondent used her family to illustrate the range of 'normality' but also utilised information from the PIL:

"Em, well I would say I don't know but by my family (laughter) they're never out of the bathroom ... I don't know what's normal at all. Em, about three, three times a week, something like that"

### Not understanding the information and confusion caused by the PILs

The CI technique illustrated that information in some of the leaflets was not understood by respondents or caused confusion. Two PILs, 'Laxatives and your constipation' and 'Your bowel and constipation', were particularly confusing and required thorough revision and simplification. This was illustrated in the following quote regarding the 'Laxatives and your constipation' PIL:

Interviewee: Before we go any further what's that word, osmotic

Interviewer: What is your understanding of the word?

Interviewee: It's Dutch to me (laughs)

Other quotes from two additional interviews illustrate a similar point:

"It's too involved for me dear, I think you should have had somebody that had a bit more brains than me."

"I don't understand the different mechanisms...the mechanism what actually it achieves I wouldn't know"

In the 'Your bowel and constipation' PIL, participants found the concept of fibre as a prebiotic confusing:

"...prebiotic, what does that mean? Something to do with antibiotics, no, .... em, I'm pretty confused with it .."

Asking the interviewee to explain a phrase highlighted issues of misunderstanding, as illustrated by this section of transcript taken from a cognitive interview using the 'Activity and constipation' PIL:

Interviewer: The first point in the leaflet says, 'being active helps prevent constipation because it stimulates gut motility'. What do you think stimulate gut motility means?

Interviewee: Again I'm afraid I'm not really familiar with the mechanics of this.

Interviewer: Okay, what does that mean to you, stimulate the gut motility?

Interviewee: I think it probably just means keeping the body mobile and free.

Interviewer: Do you mean the body or the gut?

Interviewee: Everything really.

The above section of the transcript required multiple probes and illustrates how difficult the respondent found this sentence. As a result of the CI the sentence was changed to read:

'Being active helps prevent constipation because it stimulates bowel movement.'

In the same PIL, the interviews indicated that the adjective 'vigorous' and 'moderate' when applied to 'activity' required clarification, and further examples were therefore included in the final version of the PILs of the types of activity which may fit into these categories.

"And I mean even little things, just like hanging the washing on the line it's exercise isn't it, you know e-em, I mean last night I washed all the cupboard doors in my kitchen and doing that, you know, just climbing up and down off the steps and things, that's like exercise isn't it? So if-if you just put little examples of, things like that because a lot of people wouldn't realise that that was exercise because they're just boring jobs you do every day ..."

While most respondents were familiar with the '5-a-day' message for fruit and vegetables they wanted examples of what portion size counted towards a portion. In revising the PILs, examples of portion sizes were given for commonly consumed fruit and vegetables [2].

### Levels of awareness and understanding of the condition

Within the study sample, awareness and knowledge of constipation-related factors varied greatly from very well informed individuals to those who were finding new facts about constipation within the PILs. For example, some had no knowledge about how laxatives worked whereas others appeared to have an understanding about digestion and were comfortable using nutritional terms:

"That's first of all I think you've got to chew your food properly to get it to digest properly, because if you just swallow it in chunks, your gut's going to have to work all the more breaking it down, isn't it and, making it into small pieces to get through."

"...the friendly bacteria like producing acids to stimulate and breaking down food and producing nutrients."

Reflecting on the 'Fruit and vegetables nature's laxatives' PIL, one respondent highlighted their existing awareness and readily available sources of information, mentioning the television and communication with their peers:

"I mean it is mostly things that I already knew to be quite honest whether other people would or not, but, I mean these things are sort of before your very eyes these days aren't they you get it on television and it's you know the doctor that appears on the breakfast telly as I said it's a topic of conversation the five portions a day among elderly people I'm sure"

In contrast, another respondent raised the fact that five seemed like a lot and was not exactly sure what could be included in the definition of fruit and vegetables:

"I do find five portions a lot. I don't know whether you call potatoes a vegetable, do you?"

The interview process established that some respondents thought that the information in the leaflets was not new, and in some cases was "common sense". For example, in one response, there was the assumption that people should know about the benefits of fibre in relation to constipation:

"Well I suppose it just makes you go to the toilet, it's all common sense really isn't it, don't you think it's all common sense really."

Another thought the benefits of exercise were very obvious:

"Well I should imagine anybody that's got any sense even if they are elderly you've got to have a certain amount of exercise."

This presents a challenge in developing PILs which are not patronising – yet do not assume prior knowledge. With the range of PILs produced for the LIFELAX trial, the health professional should be able to choose an appropriate leaflet dependent upon the individual patient.

There was a recurring theme of the informant not currently having constipation. Individuals perceived that, with the aid of laxatives, they were no longer constipated, although by medical definitions in terms of their need for laxatives, they are constipated:

"..... I can say honestly I'm not constipated, I was but I'm not now the thing I do take is a thing called Fybogel...."

### Additional information points

During the CI respondents suggested the addition of specific points to a number of the PILs. They recommended including advice on not getting anxious if a daily stool was not passed, also using particular foods they knew to be high in fibre.

### Problem PILs

The PIL which required the most adaptations, following CIs, was on the topic of 'Laxatives and your constipation' (which went through 3 rounds of changing and CI re-testing), followed by the PIL on 'Bowel Health'. The PIL on the overview of 'Constipation' required the least number of edits. In total, 23 changes were made across the 8 leaflets, following the use of cognitive techniques, some of which are illustrated in Table 3.

**Table 3:** Some examples of changes made across the PILs following CIs

Type of change	Before	After/action
Wording	Gut	Bowel
	Exercise	Activity
	Gut motility	Bowel movement
	Lactose intolerant	Dairy intolerance
	Probiotics	Friendly bacteria
	Bulking agents, osmotic agents, stimulants and softeners	Removed from leaflet and simplified
Examples given	Types of activity/exercise	Household activity given as an example

### Heterogeneity in the target population

The cognitive interview process uncovered the heterogeneity of this older population, with the same condition. Even with a small sample size, this process demonstrated a range of abilities, lifestyles and awareness. Some respondents were extremely active and adventurous, while others were immobile, which had to be reflected within the text of the PILs. This can be illustrated by these two quotes:

"...I go to Salsa dancing three times a week and normally I go about six or seven classes a week, if I can..."

"...oh I can be active around the house, but I cannot go for a walk, 'cause I've got no circulation in this leg..."

The PILs had to be adapted to be applicable to this range of abilities. This pilot process enabled the researchers to address this problem of heterogeneity in the target population.

The topic of laxatives was found to be very difficult and confusing as patients, understandably, were only familiar with their particular type of medication:

"I've never heard of these things, well they're enemas, senna, that's sennakot, that's another thing you can take, what's that I can't remember what that's for, no that's all things that people take I think for laxatives."

This, and other cognitive interviews, resulted in a dramatic simplification of this particular PIL.

### Layout of the leaflets

Some respondents described bullets points as being too formal and implying that the list needed to be followed. Other respondents perceived them to contribute to the overall clear layout of the leaflets, which the research team also agreed with; bullet points were therefore retained in the final version of the PILs.

Pictures were used throughout the leaflets and respondents were probed on their interpretation of these images. An image of the digestive system on the front of the 'Your Bowel and Constipation' PIL resulted in this comment:

"I mean you probably can look at that and say oh straight away what, but if you don't know a lot about the inside of your body you don't understand it do you. I mean I haven't got a, a book on, to say what that is, what that is, you probably have diagrams and all sorts of things"

The final version of this PIL had a picture of an active older person on his bicycle.

The leaflet which originally contained a 'fibre counter' and information on 'fibre and constipation' was split into two separate leaflets. The fibre counter leaflet was designed to be used as a tool for the patient to complete together with the health professional to estimate how much fibre the patient was consuming. A separate smaller informative leaflet on fibre was designed to provide 'take away' information to the patient.

## Discussion

The developmental phase of the LIFELAX trial aimed to design and produce a range of PILs to be used within the RCT. This work along with findings from earlier exploratory work from the STOOL trial established a lack of independent PILs on this topic.

As a result of the testing process with the pilot group, changes were made to the eight draft PILs. A number of changes were made to the text of the PILs and one PIL was divided into two separate PILs, resulting in nine PILs on the topic of diet, lifestyle and constipation to be used within the LIFELAX RCT.

The PILs were tested with a relatively small number of older respondents (nine) all of whom were from a similar geographical location, and were largely a culturally similar group of white, English speaking individuals. This was in keeping with the inclusion and exclusion criteria of the main trial to be conducted in the surrounding area. While these individuals were homogenous in terms of their location and cultural background, they were actually a relatively heterogeneous group in terms of lifestyle and experience of constipation. Previous work has highlighted that populations of older adults become increasingly heterogeneous [16]. While health education research tends to define older adults as a homogenous group [17], the process of testing these PILs demonstrated the variability in this age group. The PILs were changed and further designed to take into account the diversity amongst this age-group.

As these PILS were specifically designed for older adults, sensory deficits, cognitive or learning issues, hearing loss, problems with memory, reasoning and communication were considered [18]. It is also acknowledged that older adults will also need a greater amount of time for processing, learning and synthesizing new information.

While all points made by the patients were taken into account and recorded, not every comment resulted in a change to the PILs. The analysis process was extensive; both interviewers listened to the recordings of the interviews and read transcripts and noted multiple comments before deciding which factors to take into account in the final versions of the PILs. The majority of the eight original PILs were found to be generally acceptable, comprehensive and user-friendly.

Gal and Prigat's [12] exploratory work emphasises the need to pre-test PILs to establish if there are readability and comprehensibility gaps, to appreciate the contextual element of PILs and the cognitive information processes involved in understanding PILs. Cognitive interviewing techniques are flexible and can be tailored to the materials being tested. Cognitive methods have been applied to materials other than survey instruments [19], however, the use of cognitive methods in testing PILs was fairly novel and proved extremely useful for a number of reasons. Use of the probing technique, in addition to asking respondents to paraphrase, illustrated a number of important points. Asking participants to paraphrase sentences from the leaflets in their own words highlighted issues from a patient's perspective. It presented a forum in which the individuals could ask the interviewer questions about certain words, for example 'osmotic'. The technique enabled the researchers, to gain further insight into the condition of constipation, through the eyes of the target population; in particular differences in use of language between patients, researchers and health professionals in describing the condition, or even body parts. The resulting PILs used words to describe the condition which were acceptable to the patient group. The probing and paraphrasing raised areas of misunderstanding and confusion. Probing, which can be done both concurrently and retrospectively, is less burdensome than the think aloud method to the respondent, but requires more input from the interviewer. Probing can be limited by the range of the interviewers probing questions and techniques. Asking a respondent why they answered in a particular way needs to be done carefully so that criticism is not inferred. For both techniques the quality of the data will be limited by the quality of the interviewer's skills and use of techniques; poor techniques lead to poor interviews which in turn lead to poor data.

Although accepting that this age group is a diverse population, the PILs have been 'fine tuned' to their needs. While this is a time-consuming process, carrying out the cognitive interviews, enabled the development of PILs with a considerable amount of collaboration with the client group. This process of cognitive interviews could be described as time well spent in developing a set of PILs for the specific group and developing the researcher's knowledge of a condition. In comparison to this process, readability scores could be seen as an insufficient tool to be used in isolation in the development of PILs.

Dixon-Woods [20], in an analysis of publications regarding the use of PILs, identified two groups of text, those that had a patient education theme and those that were based on patient empowerment. These leaflets could be described as combining both of these by providing information (education), and empowering the patient by providing them with a reference to be used in a consultation with a health professional. When used within the Behaviour Change Counselling (BCC) arm of the LIFELAX RCT, the training provided to practitioners will advise on the use of these PILs as an information source but also a method of patient empowerment.

Further evaluation of these PILs as instruments within the RCT will be conducted at the end of the trial (2007).

## Conclusion

The process of cognitive interviewing with the target population has resulted in the production of a range of leaflets which are considered to be both understandable and useful. The process of cognitive interviewing has proved to have application in the development of PILs.

It is vital that information leaflets can be fully understood by the target population. There are a range of techniques available to test the understanding of written materials and these techniques should be employed routinely when developing patient or user information.

## Competing interests

The author(s) declare that they have no competing interests.

## Authors' contributions

AL developed the patient information leaflets with input and comments from all other authors, part of the multidisciplinary LIFELAX Team. All authors contributed to the study design. CS supervised the cognitive interviewing training of AL and AB and had input in the methodology and analysis. AB and AL conducted the cognitive interviews and analysed the data. AL, EMc, CS, BH, PM, AA and SC contributed to the drafting of this paper. All authors read and approved the final manuscript.

## Pre-publication history

The pre-publication history for this paper can be accessed here:


